# Sclerosing Angiomatoid Nodular Transformation of the Spleen: An uncommon Cause of Abdominal Pain

**DOI:** 10.5005/jp-journals-10018-1221

**Published:** 2017-05-05

**Authors:** Hakan Atas, Hakan Bulus, Göhan Akkurt

**Affiliations:** 1 Department of General Surgery, Ankara Numune Training and Research Hospital, Ankara, Turkey; 2 Department of General Surgery Kecioren Training and Research Hospital, Ankara, Turkey

**Keywords:** Abdominal pain, Sclerosing angiomatoid nodular transformation, Spleen, Splenectomy.

## Abstract

Sclerosing angiomatoid nodular transformation (SANT) is a rare and benign lesion arising from the red pulp of the spleen, with an unknown etiopathogenesis. These tumors are usually asymptomatic or cause nonspecific abdominal pain. Most SANTs are found incidentally on radiographic examination, or during surgery for an unrelated condition. Therefore, high clinical suspicion is of great importance for the diagnosis. Splenectomy provides complete cure, and no recurrence and/or malignant transformation was reported to date. In this study, a rare case of SANT was reported in a young adult male, and was discussed with the relevant literature.

**How to cite this article:** Atas H, Bulus H, Akkurt G. Sclerosing Angiomatoid Nodular Transformation of the Spleen: An uncommon Cause of Abdominal Pain. Euroasian J Hepato-Gastroenterol 2017;7(1):89-91.

## INTRODUCTION

Sclerosing angiomatoid nodular transformation (SANT) is a rare and benign lesion arising from the red pulp of the spleen, with an unknown etiopathogenesis. It was first described by Martel et al^1^ in 2004, and often affects middle-aged adults, with a slight female preponderance. These lesions are usually detected by radiological methods, such as computed tomography (CT) and magnetic resonance imaging (MRI), during the diagnostic workup of patients with chronic abdominal pain; however, definitive diagnosis requires histopathologic confirmation.^2^ Splenectomy is the treatment of choice in symptomatic patients.^3^ Herein, we report a rare case of SANT in a young adult male with long-lasting abdominal pain.

## CASE REPORT

**Fig. 1:**
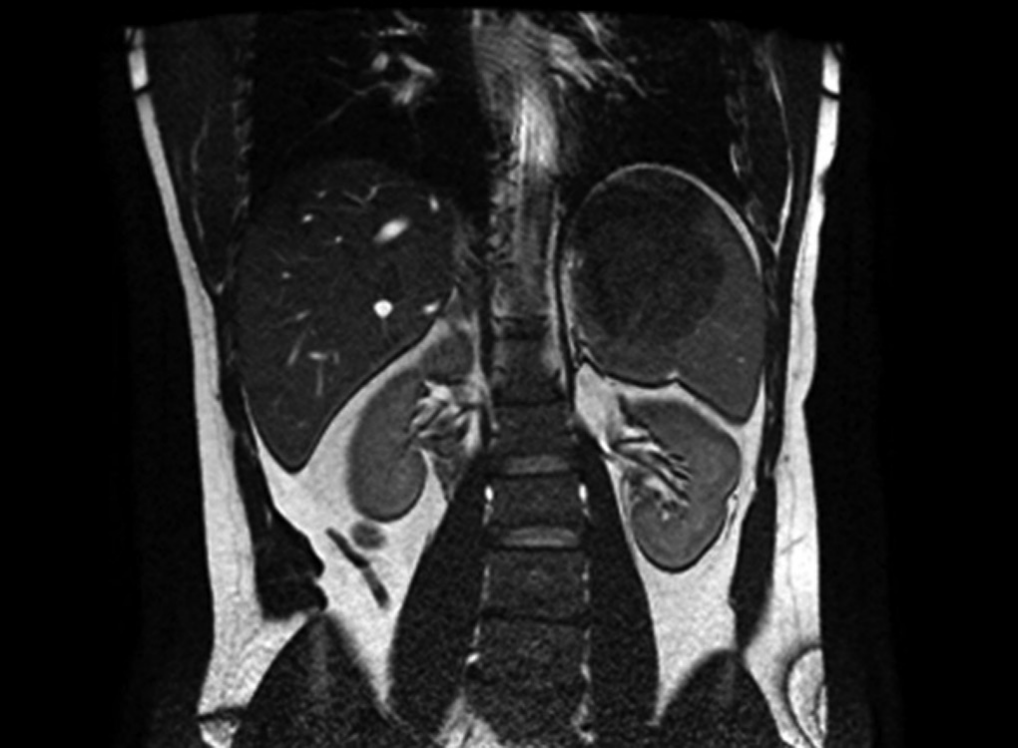
Magnetic resonance imaging of the lesion on the superior pole of the spleen

A 33-year-old male presented with left quadrant pain for 1 year. On physical examination, the spleen was palpable 4 cm below the costal margin. No symptoms of fever, weight loss, or night sweating were present. Peripheral lymph node examination was normal. Complete blood count and biochemistry parameters were all within the normal range. His medical and family history were unremarkable. A 8 cm mass located in the upper pole of the spleen was found by abdominal ultrasonography (US), without any abnormality at the other parts of the abdomen. On MRI, the lesion was isointense to parenchyma on T1-weighted sequences and mildly hyperintense on T2-weighted images. In contrast series, it also showed a heterogeneous contrast uptake during the late phase (Fig. 1).

**Fig. 2:**
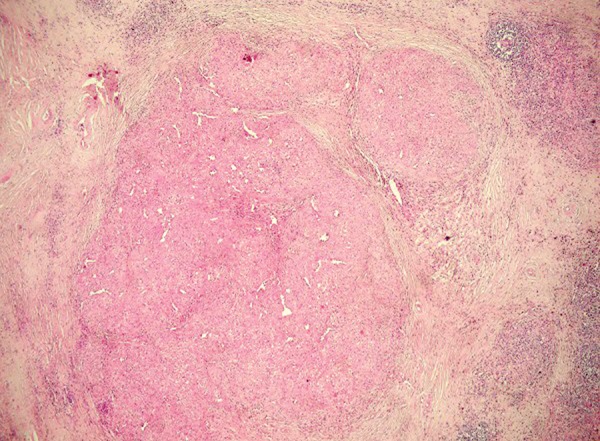
Low power photomicrograph showing variable sized nodules (H&E 40**x)**

Laparoscopic splenectomy was performed for the splenic mass. No complication occurred postoperatively, and the patient was uneventfully discharged on the 3rd day of surgery. Histopathology demonstrated hypocellular sclerotic areas along with cellular nodular regions showing increased vascularity, which comprised the vast majority of the splenectomy material (Fig. 2). According to the histopathological findings, the lesion was diagnosed as SANT. The patient was checked at regular intervals after surgery, and no sign of recurrence was detected until the first year of surgery.

## DISCUSSION

Lymphoid tumors, such as lymphoma are the most common neoplasms of the spleen, whereas nonlympho-matoid tumors were rarely reported, and are generally in vascular origin. Among those, hemangiomas, hemangio-endotheliomas, and hamartomas are the most common variants. On the contrary, SANT is an extremely rare and benign vascular lesion, which was described as multiple angiomatoid nodules embedded in a fibrosclerotic stroma, and vascular spaces surrounded by endothelial cells within each individual nodule.^1,4^ Etiopathogenesis was not clearly defined; however, several mechanisms were hypothesized to date. For instance, Martel et al^1^ suggested that angiomatoid nodules developed as a consequence of vascular obstruction. In a study by Weinreb et al,^5^ a possible association between SANT and Epstein-Barr virus was reported. More recently, Kuo et al^6^ showed that patients with SANT had elevated levels of immunoglobulin (Ig)G4 and IgG antibodies, considering IgG4-related sclerosing process in the pathogenesis of SANT. In our case, no virological and immunological association was found.

Patients with SANT are usually asymptomatic or have nonspecific abdominal pain. Thus, most SANTs are found incidentally on radiographic examination, or during surgery for an unrelated condition.^7^ Our patient suffered from intermittent mild abdominal pain for approximately 1 year, and could be diagnosed as SANT radiologically before surgery.

Radiological methods including US, CT, and MRI are useful in the diagnosis of SANT. Li et al^8^ described this tumor as an isodense appearance relative to splenic parenchyma at the late portal phase, with a calcified focus. In other studies, CT and MRI findings of SANT were reported as a solid lesion with a radial contrast uptake pattern extending from the periphery toward the center, and containing a central scar.^9^ In our case, a preliminary diagnosis of SANT was considered according to the MRI findings of the lesion. Despite the specific radiological characteristics, SANT may not be easily diagnosed preoperatively due to its rarity and being a new pathological entity. Differential diagnosis of SANT includes both benign and malignant lesions, such as hemangioma, littoral cell angioma, hemangioendothelioma, inflammatory myofibroblastic tumor, and hamartoma. Percutaneous needle biopsy is avoided because of serious complications, such as hemorrhage due to splenic rupture and needle-related seeding of tumoral cells.

These tumors should be resected in order to exclude malignancy and prevent potential risk of abdominal bleeding. Open or laparoscopic splenectomy is the mainstay surgical approach in patients with SANT, and allows complete cure. In the present case, laparoscopic splenectomy was performed for the tumor. Histopathological and immunohistochemical examinations are needed to achieve definitive diagnosis. SANT is seen as a solid, uncapsulated, red or brown nodule in the normal sized or slightly enlarged spleen, varying from 1 to 17 cm.^1,7^ SANT has a good prognosis without a risk of recurrence, and no malignant transformation was reported to date.^10^ Similarly, our patient remained well, and no evidence of recurrence was detected within the follow-up period of 1 year.

In conclusion, SANT is a rare cause of abdominal pain, and represents a diagnostic challenge. The presence of solitary splenic mass with nonspecific clinical presentations should alert radiologists and clinicians for the diagnosis of SANT. Large series are necessary for better understanding of this new pathological entity.
